# Case Report: Doubts and Pre-occupations About Being Transgender: Questioning One's Gender Identity or a Case of Obsessive-Compulsive Disorder?

**DOI:** 10.3389/fpsyt.2021.644114

**Published:** 2021-03-30

**Authors:** Elias Aboujaoude, Vladan Starcevic

**Affiliations:** ^1^Department of Psychiatry and Behavioral Sciences, Stanford University School of Medicine, Stanford, CA, United States; ^2^Faculty of Medicine and Health, Sydney Medical School, Nepean Clinical School, University of Sydney, Sydney, NSW, Australia; ^3^Department of Psychiatry, Nepean Hospital, Penrith, NSW, Australia

**Keywords:** obsessive-compulsive disorder, gender, transgender, lesbian, gay, sexual orientation, gender dysphoria, discrimination

## Abstract

We report the case of a biological female with gender identity-related doubts that were misconstrued as suggesting obsessive-compulsive disorder (OCD). The patient's parent seemed to favor an OCD explanation for the gender-based self-questioning over acceptance of possible transgender identity. We discuss what OCD is and what it is not in the context of gender identity-based doubt; analogy with the better studied sexual orientation-related OCD; cultural flashpoints around sex and gender that can become the object of OCD; how confusion about biological sex, assigned gender and gender identity can lead to clinical harm; and the role of mental health professionals in fighting the stigma faced by gender minorities.

## Introduction

Obsessive-compulsive disorder (OCD) is a heterogeneous condition almost always consisting of both obsessions and compulsions. A central phenomenon in OCD that is embedded in many obsessions and compulsions is doubting (1). Indeed, pathological doubt of the kind that would be subsumed today under OCD has been recognized since at least 1875, when Henri Legrand du Saulle published *La folie du doute avec délire du toucher* (2).

The object of OCD doubt can vary greatly—from questioning whether one has performed basic activities like locking doors to more destabilizing doubts about inadvertently causing harm or crucial aspects of one's identity such as sexual orientation. Like most other obsessions, doubts in OCD are not only unwanted and intrusive, but also ego-dystonic, i.e., alien to the person and incongruent with their perception of themselves. Such doubts are prominent in aggressive, religious and sexual obsessions, which characterize a type of OCD referred to as “unacceptable or taboo thoughts” (3). Typically more distressing than obsessions in other types of OCD (4), these obsessions are often accompanied by a sense of shame for having such obsessions, along with a need to conceal them. People will usually attempt to alleviate distress caused by these obsessions by avoiding the relevant triggers or stimuli, seeking reassurance, or performing mental compulsions.

Sexual orientation-based OCD has become a well-investigated form of OCD doubt, and is characterized by repetitive, intrusive, ego-dystonic thoughts about sexual orientation, often accompanied by reassurance seeking, and ritualistic verification of a patient's sexuality. More recently, gender identity has become a topic of OCD doubt. As this case illustrates, though, not all gender identity-based doubt is OCD. Clinicians should be attuned to the presenting nuances and subtleties to determine what does and does not constitute psychopathology when it comes to this type of doubt.

## Case Presentation

Ms. M. is an 18-year-old white biological female, a high school senior with no prior history of psychiatric care, who sought an evaluation in our clinic partially at the urging of her father. She requested that we use female pronouns to address her. We met with Ms. M in person, and, with her consent, we spoke with her father over the phone.

According to Ms. M's father, Ms. M. would “obsess” about particular themes starting in infancy. Between ages 1 and 3, she fixated on red objects; she would cry for hours until she was handed one. Ms. M's father stated that in kindergarten, Ms. M became “obsessed” with Pixar's Ratatouille movie, which featured a rat that dreamt of becoming a chef; she would watch it daily and “constantly” talked about it. Eventually, Ms. M's interest shifted to Super Mario 3D World, a video game she would play “incessantly.” The highly engaging, narrow interest would change every 1–2 years into adolescence, and, when at age 16, Ms. M first mentioned to her family that she had been preoccupied with gender identity-related doubt, her father assumed it was “another obsession.”

Ms. M. reported first crossing traditional gender roles at age 8. She would steal her father's razor and shaving supplies and engage in enjoyable solitary play in the bathroom, trying to shave her non-existent facial hair in front of the mirror. The behavior continued for ~3 years until she got caught after accidentally cutting herself. In high school, Ms. M. started questioning her assigned gender more explicitly, wondering whether she might feel more fulfilled as a male. Her doubts grew to daily preoccupations as the disconnect she felt between her assigned gender and the one she identified with became increasingly distressing. Imagining herself adopting typically male social roles and physical traits was comforting and made her feel “at peace.” She did not feel compelled to do anything to alleviate the gender identity doubts, such as checking to make sure she is female or seeking reassurance about it. Her menstrual cycle and secondary female sexual characteristics like breast tissue and rounded hips became sources of discomfort for her. She described going from doubting whether she might identify more with being male to believing she was male but doubting whether society would accept her as such. As she increasingly researched gender issues online, she found support in transgender community forums and developed the courage to share her struggle with her parents.

Ms. M. completed the Yale-Brown Obsessive-Compulsive Scale (Y-BOCS) checklist (5) in clinic, which revealed that Ms. M was at times bothered by asymmetrical arrangements but was not driven to rearrange her environment. She also experienced rare intrusive, bothersome thoughts about inadvertently harming her siblings, without any history of aggression and associated neutralizing ritual or avoidance. She expressed some superstitions around the number 13, without associated compulsions or avoidance. Together, the symmetry, harm and superstition concerns occupied <30 min daily and did not cause significant distress or functional impairment, thereby failing to support the diagnosis of OCD. Her Y-BOCS score was 7.

Ms. M. described close relationships, including with her parents, two brothers, and three lifelong friends. She has enjoyed superior grades in high school and has just been accepted to college, where she intends to major in psychology. She has never been sexually active and describes being physically attracted to females.

Ms. M. seemed relieved to hear that her gender identity-based doubts were not consistent with OCD, despite the existence of subclinical OCD symptoms unrelated to gender. She declined a referral to a clinic that specializes in providing support around gender identity issues, fearing that its providers might “push” her toward coming out as transgender before she was ready to do so. Ms. M. did, however, agree to seek out a general therapist for supportive counseling. As no clinical diagnosis was made, Ms. M. was not scheduled for a return visit to the specialty OCD clinic where we saw her, and we have no information on whether she subsequently pursued seeing a general therapist.

Our conceptualization of Ms. M's gender-related doubts did not entirely satisfy her father, however, who viewed Ms. M's gender identity questioning as a likely transient obsession that will subside over time. Interpreting the situation as a “treatable” OCD crisis seemed easier than the possibility of having a transgender child.

## Discussion

Sexual orientation-related obsessions might serve as a “template” for understanding how transgender identity can also be a topic of OCD-related doubt. Obsessions about sexual orientation are often “classified” as unacceptable or taboo obsessions because they load predominantly onto the corresponding factor in factor analytic studies (6). For heterosexual patients, they are posited to include recurrent doubts about whether one is gay; unwanted and intrusive fears of being or becoming gay or of others thinking that one is gay; and intrusive, unwanted mental images of gay sexual behavior (7–9). Compared to patients with OCD who do not have sexual orientation-related obsessions, those with such obsessions report significantly more distress and interference from obsessions, as well as greater proneness to avoidance (8). Various responses to sexual orientation-related obsessions have been described, including seeking interpersonal reassurance; self-reassurance; avoidance of the specific situations that might provoke these obsessions; behaviors that “test” one's sexual orientation; need to confess to others; and mental compulsions (7, 9, 10). The purpose of these behaviors is to alleviate distress and attain certainty by “proving” to the person that he or she is not gay. However, as is usually the case with OCD, relief from compulsions and other attempts to alleviate obsession-induced distress is only temporary, and the obsessions typically return.

Obsessions related to gender identity may resemble sexual orientation-related obsessions, except for their content. While assigned gender is based on the person's natal genitalia, gender identity is a category of social identity and refers to the person's deeply held identification as male, female, some of both, or neither, and does not always align with biological sex (11). Similar to, but perhaps more than, gay sexual orientation, the rejection of one's assigned gender identity remains a cultural taboo. This makes it fertile ground for OCD, since OCD often involves the fear of breaking cultural norms and dictates. Some published reports in the literature can help inform the approach to a case as the one we describe here. In one report of OCD with gender identity-related obsessions, Safer et al. (12) described a male patient with ego-dystonic doubts about being transgender that were accompanied by various mental compulsions (e.g., “testing” his reactions to certain thoughts or images) and reassurance seeking about his “true” gender identity. Similarly, Uvais et al. (13) described a “remittable form of gender identity disorder” in a 24-year-old male with gender identity-related doubt who was diagnosed with OCD and treated successfully with fluoxetine and risperidone.

While obsessions are frequently accompanied by doubt, not all instances of doubting are associated with obsessions and OCD. Individuals who question their gender identity may well have doubts about how their biological sex and gender identity interface, feel anxious about flouting gender-based conventions, and worry about discrimination. However, the possibility of embracing the gender that they feel is more aligned with who they are can bring comfort to such individuals. In OCD, gender questioning is a destabilizing, ego-dystonic thought that lacks positive charge and is more likely to result in repetitive checking to “make sure” that the person still identifies with their assigned gender. Indeed, ego-“dystonicity” may be a crucial element in differentiating “normal” gender questioning from gender questioning as OCD symptom.

Ms. M. did question her assigned gender and worried whether she would be accepted by society if she lived a traditionally male gender role, but these concerns and doubts were not experienced as unwanted, intrusive, or ego-dystonic. To the contrary, Ms. M has since childhood drawn satisfaction from gender non-conforming behaviors and now finds peace when she imagines embracing a male gender identity. In addition, Ms. M. did not report that she had to “do something” with her concerns in order to experience relief or “prove” to herself that she was not a male, such as seek reassurance or perform another compulsion. Therefore, Ms. M's questioning of her assigned gender, and what it would be like if she were male, do not represent obsessions. Instead, they are better understood as a genuine quest for her true gender identity. Indeed, even if she may be seen to have symptoms of subclinical OCD based on symmetry, harm and superstition concerns, the absence of both ego-dystonic obsessions about being transgender and compulsions in response to such obsessions does not support a gender identity-based diagnosis of OCD.

Recent years have brought greater understanding to gender identity as a non-binary, non-static construct that should not exclusively be determined by biological sex or limited by rigid norms and expectations. However, acceptance of this insight has proven to be an uphill battle due to societal prejudice. Encouraging milestones lend hope that gender non-conforming individuals will face fewer obstacles in the future; in 2020, for example, the US Supreme Court ruled that discrimination against transgender individuals would violate the Civil Rights Act (14). Nevertheless, much stigma remains, as perhaps manifested in the reaction by Ms. M's father, who found a psychiatric diagnosis for his daughter—itself a subject to stigma—more acceptable than the possibility of her identifying as male. Unfortunately, this tendency to assume that *any* concern or doubt about one's gender identity reflects psychopathology is not rare. To some extent, the psychiatric profession is also responsible, given the diagnostic categories of transsexualism and gender identity disorder used in previous iterations of the Diagnostic and Statistical Manual of Mental Disorders (DSM). Even the DSM-5 diagnostic category of gender dysphoria (15) has been criticized (16), not least because it can be seen to pathologize some expected distress among individuals questioning their assigned gender identity. In that sense, a small improvement has been made by the decision to avoid the term “gender dysphoria” in the 11th Revision of the International Classification of Diseases (ICD-11) and to instead refer to “gender incongruence” and not classify it as a mental disorder (17). Whether gender incongruence should be classified as a “condition related to sexual health” in ICD-11 is a matter of yet another debate.

Regardless of the debates about understanding and conceptualizing doubts and concerns related to gender identity, mental health professionals often need to navigate these complex issues. Therefore, mental health professionals play a critical role in de-pathologizing such doubts and concerns when they are not better explained by a mental disorder. It is important to recognize that clinicians can cause harm if they are not attentive to the nuances distinguishing biological sex, assigned gender and gender identity, and if they are untrained to make appropriate assessment. Mental health clinicians are also called upon to model acceptance, thereby helping enhance the safety and well-being of a community that has long been shown to be disproportionately impacted by many hardships, including violence (18). To that end, national advocacy groups such as the Gay and Lesbian Alliance Against Defamation (19) offer valuable guidelines that clinicians would do well to follow, such as the adoption of inclusive language; respect for privacy by not “outing” someone's transgender identity without their permission; eschewing references to “real name” or surgical status in discussions with or about transgender individuals; and raising awareness of the prejudice and hate crimes plaguing the community.

Finally, diagnosing doubt-based OCD can be challenging even for seasoned clinicians with considerable OCD expertise. When gender or other socio-culturally charged topics is a focus of OCD, the process can become even more challenging (20). In such situations, clinicians should not feel deterred from consulting with colleagues or seeking a “second opinion” from an OCD specialist.

## Conclusion

Intrusive, unwanted and ego-dystonic doubts and preoccupations are a hallmark of OCD. In contrast, doubts and preoccupations that may be distressing but are not experienced as intrusive, unwanted, and ego-dystonic should not be automatically attributed to OCD or any psychopathology. Moreover, doubts and preoccupations in OCD are intolerable and the person typically attempts to cope with them via compulsions and/or excessive reassurance seeking or avoidance; such coping with doubts and preoccupations usually does not occur in the absence of OCD. These distinctions are particularly important when doubts and preoccupations pertain to crucial aspects of one's identity, including a possibility of being transgender. Mental health professionals play a central role in distinguishing between OCD with gender identity-related obsessions, and the questioning of one's gender identity that is based on the deeply felt incongruence between assigned gender and the gender that the person identifies with. Ability to conduct proper assessment in this regard is not only important in terms of determining whether or not a mental disorder is present, but it has far-reaching implications for the minimization of stigma and discrimination that often accompany both people with mental disorders and transgender individuals.

## Data Availability Statement

The original contributions presented in the study are included in the article/Supplementary Material, further inquiries can be directed to the corresponding author/s.

## Ethics Statement

Written informed consent was obtained from the individual(s) for the publication of any potentially identifiable images or data included in this article.

## Author Contributions

EA and VS contributed to conceptualizing, researching, writing, and editing this manuscript.

## Conflict of Interest

The authors declare that the research was conducted in the absence of any commercial or financial relationships that could be construed as a potential conflict of interest.
